# Supplementary material for: Production of exopolysaccharide by strains of Lactobacillus plantarum YO175 and OF101 isolated from traditional fermented cereal beverage
Source: PeerJ. 2018 Oct 10;6:e5326. doi: 10.7717/peerj.5326 (PMC6186159; doi:10.7717/peerj.5326)
Supplement: Supplemental Information 1 — TLC and dry EPS samples. [file peerj-06-5326-s001.docx]

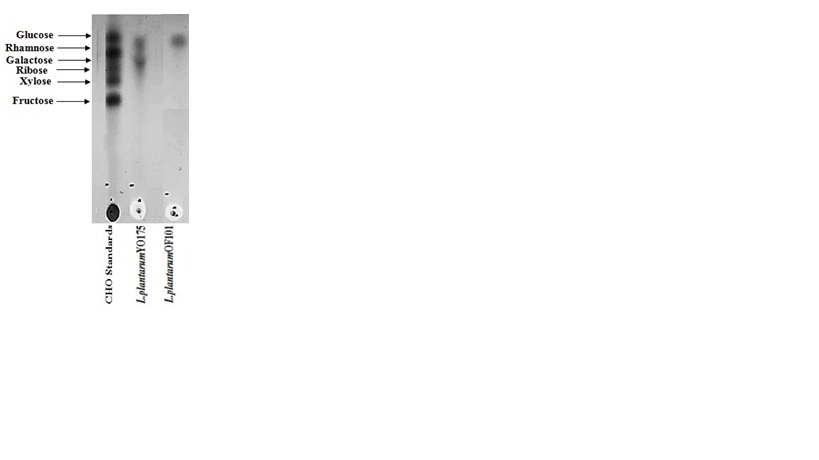


**FigS1. TLC plate showing the monosaccharide composition of the EPS samples**


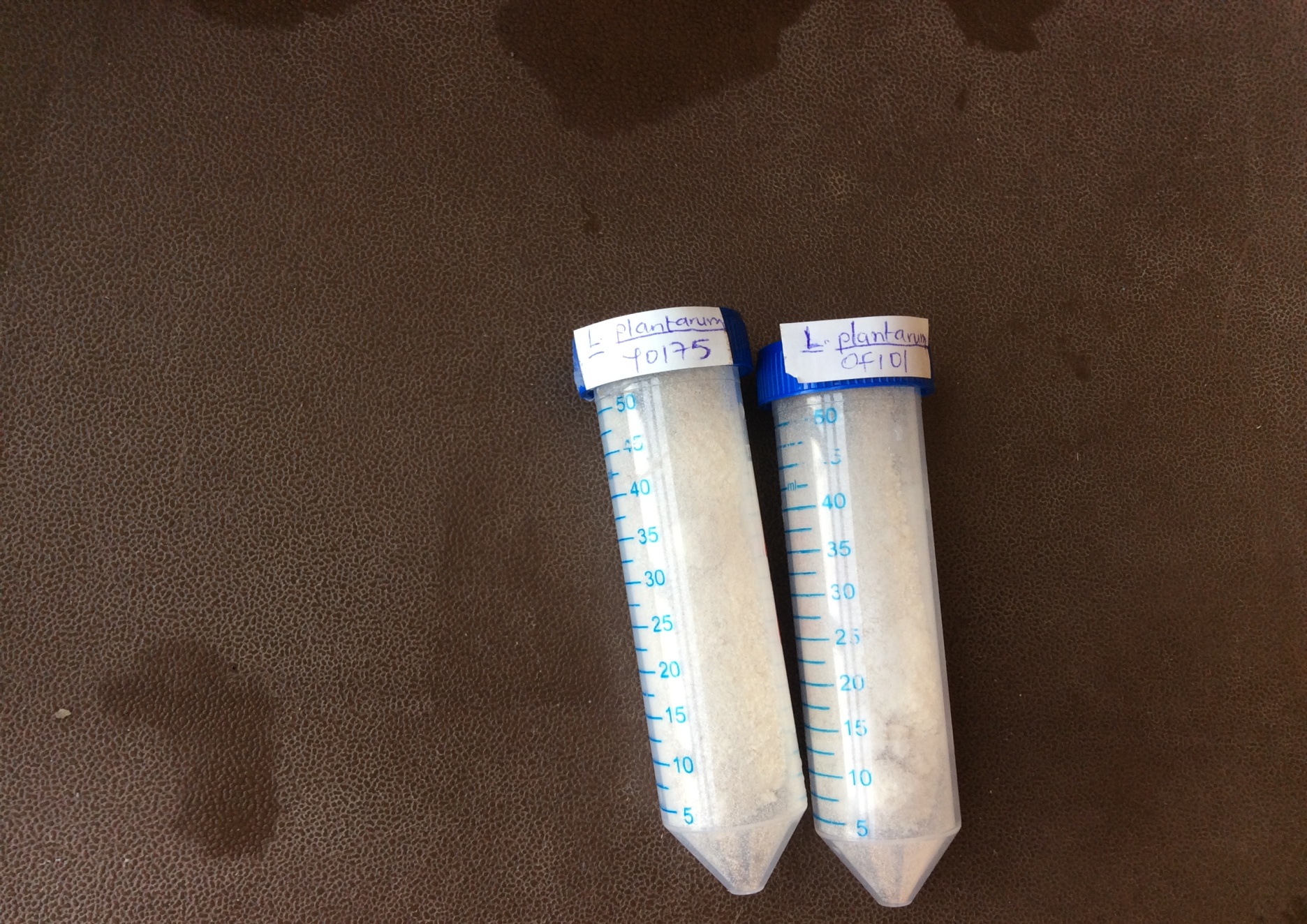


**FigS2. Dry EPS produced on MRS-Sucrose modified media**
